# Up- and Down-Regulation of Enzyme Activity in Aggregates with Gold-Covered Magnetic Nanoparticles Triggered by Low-Frequency Magnetic Field

**DOI:** 10.3390/nano14050411

**Published:** 2024-02-23

**Authors:** Maxim M. Veselov, Maria V. Efremova, Andrey N. Prusov, Natalia L. Klyachko

**Affiliations:** 1 School of Chemistry, Lomonosov Moscow State University, Moscow 119991, Russia; veselov.mac@gmail.com (M.M.V.); m.efremova@tue.nl (M.V.E.); 2 Department of Applied Physics and Science Education, Eindhoven University of Technology, 5600 MB Eindhoven, The Netherlands; 3 A.N. Belozersky Institute of Physico-Chemical Biology, Lomonosov Moscow State University, Moscow 119992, Russia; prusov@genebee.msu.su

**Keywords:** magnetic nanoparticles, low-frequency magnetic field, enzyme regulation

## Abstract

The modern global trend toward sustainable processes that meet the requirements of “green chemistry” provides new opportunities for the broad application of highly active, selective, and specific enzymatic reactions. However, the effective application of enzymes in industrial processes requires the development of systems for the remote regulation of their activity triggered by external physical stimuli, one of which is a low-frequency magnetic field (LFMF). Magnetic nanoparticles (MNPs) transform the energy of an LFMF into mechanical forces and deformations applied to enzyme molecules on the surfaces of MNPs. Here, we demonstrate the up- and down-regulation of two biotechnologically important enzymes, yeast alcohol dehydrogenase (YADH) and soybean formate dehydrogenase (FDH), in aggregates with gold-covered magnetic nanoparticles (GCMNPs) triggered by an LFMF. Two types of aggregates, “dimeric” (with the enzyme attached to several GCMNPs simultaneously), with YADH or FDH, and “monomeric” (the enzyme attached to only one GCMNP), with FDH, were synthesized. Depending on the aggregate type (“dimeric” or “monomeric”), LFMF treatment led to a decrease (down-regulation) or an increase (up-regulation) in enzyme activity. For “dimeric” aggregates, we observed 67 ± 9% and 47 ± 7% decreases in enzyme activity under LFMF exposure for YADH and FDH, respectively. Moreover, in the case of YADH, varying the enzyme or the cross-linking agent concentration led to different magnitudes of the LFMF effect, which was more significant at lower enzyme and higher cross-linking agent concentrations. Different responses to LFMF exposure depending on cofactor presence were also demonstrated. This effect might result from a varying cofactor binding efficiency to enzymes. For the “monomeric” aggregates with FDH, the LFMF treatment caused a significant increase in enzyme activity; the magnitude of this effect depended on the cofactor type: we observed up to 40% enzyme up-regulation in the case of NADP^+^, while almost no effect was observed in the case of NAD^+^.

## 1. Introduction

The global trends toward more sustainable processes and biotechnological routes for obtaining chemical products have contributed to the increased use of enzymes in the industrial field [1,2]. Industrial processes based on highly active, selective, specific, biodegradable, and renewable enzymatic reactions are fully adjustable to “green chemistry” principles [3,4]. However, the effective application of enzymes in industrial processes requires the development of systems for the remote regulation of their activity triggered by external physical stimuli [5,6,7]. Because changes in an enzyme’s structure determine changes in its activity, this approach can be the basis for a universal method of enzyme regulation. Berezin and co-authors showed the first example of such regulation in the late 1970s [8]. According to their study, immobilized chymotrypsin (ChT) molecules were stressed by mechanical forces applied by stretching the enzyme carrier, and, as a result, the enzyme activity decreased. Later, the regulation of an enzyme’s activity by changing its conformation was shown using the light-sensitive isomerization of the molecules bound to the enzyme [9,10,11,12] or included in enzyme carriers [13,14]; changes were also induced by microwave radiation [15,16], thermo-sensitive changes in hydrogel carriers [17], or changes in the local temperature induced by a magnetic field [18].

We have previously shown that the activity of trypsin [19] and ChT [19,20,21,22] bound to the surface of magnetic nanoparticles (MNPs) was remotely down-regulated under exposure to a low-frequency magnetic field (LFMF). The so-called magneto-nanomechanical (MNM) effect [23] is a result of rotational motions of MNPs in the LFMF that lead to the generation of forces and deformations applied to enzyme molecules that change their secondary structures [22]. The nature and magnitude of such forces vary depending on the composition of the MNP–enzyme aggregate. In “dimeric” aggregates (aggregates in which the enzyme molecules are bound to several MNPs simultaneously), the application of an LFMF generates stretching, compressing, twisting, and shifting forces at a magnitude of up to 100 pN [24]. When using the term “dimeric” aggregate, we mean an aggregate that consists of two or more particles (GCMNPs) conjugated to each other via enzyme molecules. In contrast, in “monomeric” aggregates (aggregates in which the enzyme molecules are only bound to one MNP), the application of an LFMF generates weak (amounting to only several pN) hydrodynamic forces [23]. Here, we present the up- and down-regulation of two biotechnologically important enzymes: yeast alcohol dehydrogenase (YADH) and soybean formate dehydrogenase (FDH). We used these two redox enzymes as both are NAD(P)^+^-dependent, well-known and similar to each other in terms of general properties, and frequently used in industrial processes based on enzymatic reactions. Previously, we demonstrated the possibility of the down-regulation of the hydrolytic enzyme ChT [19], so by using YADH and FDH, two enzymes with different structures and activity, we are expanding the number of enzyme examples to demonstrate the universality of the MNM approach. Moreover, the *Glycine max* enzyme has a special feature—a very small but detectable level of activity with NAD(P)^+^ together with normal NAD^+^ activity. We produced aggregates consisting of YADH or FHD and gold-covered magnetic nanoparticles (GCMNPs) and studied the effect of an LFMF on the activity of the enzymes in the aggregates. We observed different effects of the LFMF on the enzyme activity depending on the MNP–enzyme aggregate type. Also, we discuss the major principles and limitations related to the up- and down-regulation of enzyme activity.

## 2. Materials and Methods

### 2.1. Materials

Iron (II) chloride tetrahydrate (FeCl_2_·4H_2_O, 98%), iron (III) chloride (FeCl_3_), gold (III) chloride trihydrate (HAuCl_4_·3H_2_O), sodium citrate trihydrate (Na_3_C_6_H_5_O_7_·3H_2_O), citric acid (C_6_H_8_O_7_), hydrochloric acid (HCl, 37%), ammonium hydroxide solution (NH_3_·H_2_O, 29%), perchloric acid (HClO_4_, 70%), lipoic acid (LA) (C_8_H_14_O_2_S_2_, 99.9%), bifunctional thiol- and carboxylic-group-modified polyethyleneglycol with a molecular mass of 5 kDa (SH-PEG_5000_-COOH), N-(3-dimethyl aminopropyl)-N′-ethyl carbodiimide hydrochloride (EDC), sulfo-N-hydroxysuccinimide (S-NHS), tris(hydroxymethyl)aminomethane (Tris), ethanol (C_2_H_5_OH), β-Nicotinamide adenine dinucleotide hydrate (NAD^+^) >99%, β-Nicotinamide adenine dinucleotide phosphate sodium salt hydrate (NADP^+^) >99%, and sodium formate (CH_2_O_2_) were all purchased from Sigma-Aldrich (St. Louis, MO, USA). YADH was purchased from Reachim (Budapest, Hungary). FDH from *Glycine max* was kindly provided by Prof. Tishkov from Lomonosov Moscow State University (Moscow, Russia). The expression and purification procedures can be found in [25]. Deionized (DI) water (18.2 MΩ·cm Werner Easypure II system, Leverkusen, Germany) was used in all experiments.

### 2.2. GCMNPs Synthesis, Surface Modification, and Enzyme Conjugation

GCMNPs were synthesized using a previously described two-step procedure [21,22]. The magnetic core was synthesized via the co-precipitation of Fe^2+^ and Fe^3+^ salts and then covered with a gold shell via the citrate reduction of HAuCl_4_. The reaction mixture was centrifugated to separate non-covered nanoparticles. For surface functionalization, 10 mL of purified GCMNPs dispersed in citrate buffer was mixed with 10 mL of 1 mg/mL LA or SH-PEG_5000_-COOH solution and stirred overnight at room temperature. Afterward, the product was dialyzed three times against 1 L of DI H_2_O for 6 h. The conjugation of YADH and FDH to functionalized GCMNPs was performed via a two-step procedure involving the activation of carboxylic groups of LA on the surfaces of GCMNPs with EDC and S-NHS, followed by the formation of amide bonds between LA and YADH (for details, see Table S1).

### 2.3. Enzymatic Activity

The activity of YADH and FDH in aggregates with GCMNPs was studied using UV–VIS spectroscopy conducted at 25 °C, measuring the increase in the NADH (or NADPH) concentration at 340 nm during the ethanol (in the case of YADH) and formate (in the case of FDH) oxidation catalyzed by the enzyme. A cuvette containing the suspension of the conjugated enzyme in a 20 mM Tris-HCl buffer (pH 8.2) was placed into an LFMF generator with a temperature control (TOR 01/12, Nanodiagnostica LLC, Tambov, Russia). The sample was exposed to three cycles of a “pulsed” LFMF (*f* = 50 Hz, *B* = 140 mT, Pulse/Pause = 1 min/30 s). The temperature during the field exposure remained constant within a ∼0.1 K precision of the temperature measurement. The control samples were not exposed to an LFMF. After that, the cofactor (NAD^+^ or NADP^+^) solution and ethanol or sodium formate for YADH and FDH, respectively, were added to the cuvette, and the kinetic curves were recorded for 5 min using a SpectraMax M5 (Molecular Devices, San Jose, CA, USA) UV–VIS spectrometer (or using NanoDrop One C (Thermo, Waltham, MA, USA) in the case of microsamples). In some cases, to study the effect of cofactor presence or absence on the effect of an LFMF, NAD^+^ was added before LFMF exposure. Changes in NADH concentration were determined using an extinction coefficient: ε = 6200 M^−1^ cm^−1^ [26]. The initial rate (R) of enzymatic reaction was calculated as a change in product (NAD(P)H) concentration over time. To present the changes in enzyme activity as a result of LFMF exposure, the R value after LFMF treatment was normalized to the R value before LFMF treatment.

### 2.4. Sample Characterization

A transmission electron microscope, JEM 1400, 120 kV (JEOL, Tokyo, Japan), was used to examine the size and morphology of the MNPs. Samples for transmission electron microscopy (TEM) were dropped onto a copper 200-mesh grid and dried. The average MNP’s size was determined by analyzing at least 50 particles using ImageJ software version 1.52 (National Institutes of Health, USA). The determination of hydrodynamic size via Nanoparticle Tracking Analysis (NTA) was carried out using a NanoSight NS500 instrument (Malvern Panalytica, Malvern, UK) equipped with an 80 mW 532 nm laser. The size distribution of the MNPs was determined using NanoSight 2.3 software (Malvern, UK). The zeta-potential of MNPs was measured via dynamic light scattering (DLS) using a ZetaSizer Nano ZS (Malvern, UK), averaging 20 runs per measurement using the Smoluchowski model. Samples for DLS were dissolved in a 10 mM KCl solution, and measurements were taken using a transparent zeta-potential cell (DTS1060C). Mössbauer spectra of ^57^Fe nuclei at room temperature were recorded with an MS-1104Em spectrometer (Southern Federal University, Research Institute of Physics, Rostov-on-Don, Russia) in transmission geometry with an α^57^Co(Rh) radiation source. Spectral analysis was performed in the Univem MS 701 program, and the relative intensities (area) of the initial spectra were determined.

## 3. Results and Discussion

### 3.1. GCMNP Synthesis and Functionalization

The GCMNPs were synthesized using a two-step procedure [21,22] based on, first, the co-precipitation of Fe^2+^ and Fe^3+^ ions to form the magnetic cores of iron oxide and, second, covering them with a gold shell via the reduction of HAuCl_4_. The gold shell surrounding the magnetic core provided the GCMNPs colloidal stability and the ability to be further surface-modified with sulfide or disulfide ligands. According to the TEM results, the mean diameter of the iron oxide cores was 9 ± 2 nm, while the mean diameter of the GCMNPs was 25 ± 3 nm (Figure S1). The magnetite/maghemite ratio for the synthesized iron oxide magnetic core was 2:1 based on the analysis of Mossbauer spectra (Figure S2 and Table S2). The rotational motion (called Brownian relaxation) of MNPs under exposure to an LFMF is observed only for MNPs with a radius greater than the critical radius, *R_C_* [22,23]. For magnetite and maghemite, the *R_C_* values were ~4.1 and ~5.9 nm, respectively [22]. Based on the diameter and phase composition of the magnetic cores, we supposed that the synthesized GCMNPs could undergo mechanical rotation, which is required for the MNM approach.

The surface of the GCMNPs was functionalized with two types of thiol-containing ligands: a low-molecular-mass ligand, lipoic acid (GCMNP-LA), or a high-molecular-mass ligand, SH-PEG_5000_-COOH (GCMNP-PEG). Because the interaction between sulfur and gold is very strong (the strength of a S–Au bond is 48 kcal/mol [27]), we believe that such ligands may effectively cover the gold surface of GCMNPs. The functionalized GCMNPs were negatively charged; according to DLS measurements (Figure S3), the average zeta-potentials were −30 ± 7 mV and −10 ± 1 mV for GCMNP-LA and GCMNP-PEG, respectively. According to NTA analysis (Table 1 and Figure S3), the mean hydrodynamic diameters were 52 ± 1 nm and 74 ± 1 nm for GCMNP-LA and GCMNP-PEG, respectively. It should be noted that NTA showed a single peak in the size distribution of the functionalized GCMNPs, indicating the low polydispersity of the MNPs.

### 3.2. Enzyme Conjugation to the GCMNPs’ Surfaces

The conjugation of the enzyme molecules to the modified GCMNPs was performed via a two-step procedure to prevent the enzyme molecules from cross-linking without nanoparticles. We have previously shown that the activity of ChT in “dimeric” aggregates with GCMNPs was down-regulated under exposure to an LFMF. To maximize the effect of the LFMF and maximize the number of “dimeric” aggregates, we varied the enzyme conjugation conditions (using YADH as an example), such as the enzyme and cross-linking agent (EDC/S-NHS) concentrations (see the Section 2 and Supplementary Materials). We have previously shown that enzyme down-regulation in “dimeric” aggregates subjected to an LFMF treatment was much more substantial with short low-molecular-weight ligands [20]; thus, we used GCMNP-LA in the present work. The mean hydrodynamic diameter of the aggregates produced at various YADH and cross-linking agent concentrations (samples D1–D4) was ~2–3 times larger than that of the GCMNPs unmodified with the enzyme (Table 1). Moreover, for all of the aggregates, the position of the main peak shifted to a higher value, and multiple peaks appeared in the distribution pattern (Table 1 and Figure S4). Such changes in the hydrodynamic size distributions can indicate the formation of aggregates consisting of several MNPs [22,28]. Also, we previously verified the formation of “dimeric” aggregates with chymotrypsin using TEM [22]. The hydrodynamic diameter of such aggregates was also 2–3 times greater than that for GCMNPs without the enzyme. It was shown in [28] that the hydrodynamic diameter of dimeric aggregates was at least 1.5 times greater than that of monomeric nanoparticles. Accordingly, we believe that the similar changes in the hydrodynamic diameters of aggregates obtained in this work also indicate the formation of aggregates consisting of at least two GCMNPs. It should be pointed out that varying the concentration of YADH and EDC/S-NHS did not lead to changes in the hydrodynamic size distributions of the synthesized aggregates.

Hydrodynamic size distributions were also studied for the GCMNP-LA aggregates with FDH (sample D5, Table 1, and Figure S4). In this case, the mean hydrodynamic diameter of the aggregates was 2.4 times larger than that of the MNPs that were not modified with the enzyme. We also observed the appearance of several peaks in the distribution pattern for the GCMNP-LA aggregates with FDH as well as for the GCMNP-LA aggregates with YADH. Thus, based on the NTA analysis, we believe we synthesized GCMNP-LA aggregates with YADH and FDH with a “dimeric” structure.

For the synthesis of “monomeric” aggregates with FDH, we used GCMNP functionalized with SH-PEG_5000_-COOH (GCMNP-PEG), considering that a PEG shell can increase MNPs’ colloidal stability and promote the formation of “monomeric” aggregates. The synthesis mechanism of “monomeric” aggregates was generally the same as that for “dimeric” aggregates (the formation of amide bonds between amino groups of the enzyme and carboxylic groups of the GCMNPs). However, to synthesize “monomeric” aggregates, we used a 3.2-fold-higher enzyme concentration than that for the production of the “dimeric” aggregates. Thus, the ratio of amine groups on the surface of the enzyme to the carboxylic groups on the GCMNPs’ surface was higher in the case of the “monomeric” aggregates, and, as a result, the formation of “monomeric” aggregates was preferential. Using HS-PEG5000-COOH, we also increased the amino-to-carboxylic-groups ratio in comparison with GCMP-LA (zeta-potential measurements, Figure S3a,b), which further promoted the formation of “monomeric” aggregates. As a result of FDH conjugation to GCMNP-PEG, the pattern of size distribution revealed through NTA remained unchanged (Figure S5a). We observed only a slight decrease in nanoparticle concentration (probably due to nanoparticle loss during the purification step) as well as a low percentage (<5%) of relatively large (more than 125 nm in diameter) nanoparticles. The mean hydrodynamic diameter of the GCMNP-PEG aggregates with FDH (sample M1) was 97 ± 2 nm, i.e., only 23 nm larger than that of the MNPs that were not modified with the enzyme. Thus, we can say that sample M1 indeed contains “monomeric” aggregates; their formation was also confirmed via TEM (Figure S5b).

**Table 1:** Mean hydrodynamic diameters, peak positions, and concentrations of functionalized GCMNPs and GCMNPs–enzyme aggregates observed using NTA. The position of the main peak in the distribution pattern is indicated in bold. The mean hydrodynamic diameter values are presented as the Mean ± SEM (=SD/√*N*, where *N* is the value of completed nanoparticle tracks during the measurement).

Sample	Mean Hydrodynamic Diameter, nm	Peak Positions, nm
GCMNP-LA	52 ± 1	**41**
GCMNP-PEG	74 ± 1	**68**
D1	147 ± 3	75, **93**
D2	158 ± 3	62, **108**, 146, 329
D3	104 ± 2	**74**, 302
D4	159 ± 4	**92**, 171, 240
D5	127 ± 2	**72**, 191
M1	97 ± 2	**62**

### 3.3. Down-Regulation of Enzymes in “Dimeric” Aggregates

We examined the effect of LFMF exposure on the catalytic activity of enzymes in aggregates with MNPs. The initial rate (*R*) of Reaction 1 and Reaction 2 used to determine YADH and FDH activity, respectively, was monitored according to product (NADH or NADPH) accumulation.

(1) $$CH_{3}CH_{2}OH+NAD^{+}\to CH_{3}CHO+NADH+H^{+}$$

(2) $$HCOO^{-}+NA{D(P)}^{+}\to CO_{2}+NAD(P)H$$

The impact of the LFMF on the activity of YADH and FDH in the aggregates with MNPs was determined using post-effect mode when substrates (NAD^+^/NADP^+^ as well as ethanol or sodium formate in the case of YADH or FDH, respectively) were added to the aggregates after LFMF treatment. It should be noted that both YADH and FDH have broad pH optima: 8–9 for YADH (with a maximum at 8.1, approximately [29]) and 6–9 for FDH [30]. The optimal temperature for YADH is 30 °C [29], while that for FDH is 55 °C [31]. It should be pointed out that the heat generated by the GCMNPs under an LFMF is negligible, and measuring the activity of enzymes at 25 °C allows one to avoid any thermal inactivation and denaturation for both enzymes.

**Figure 1:**
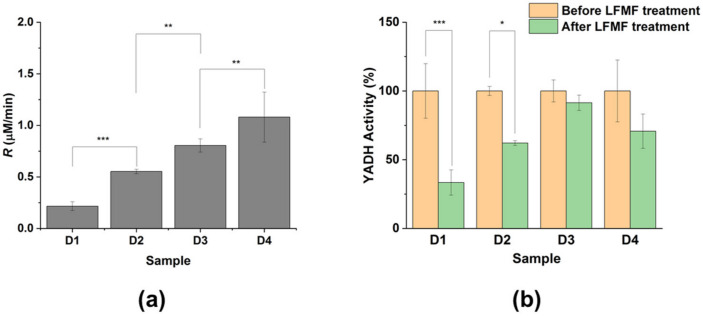
The effect of three cycles of a “pulsed” LFMF (*f* = 50 Hz, *B* = 140, Pulse/Pause = 1 min/30 s) on the enzymatic activity of YADH in “dimeric” aggregates with GCMNP-LA. (**a**) The initial rate (*R*) of Reaction 1 catalyzed by samples (D1–D4) synthesized at different concentrations of YADH and EDC/S-NHS. (**b**) Changes in YADH activity as a result of LFMF treatment. The concentration of aggregates was 1 × 10^10^ particles/mL. The following conditions apply for the results shown: 20 mM of Tris-HCl buffer (pH 8.2), 9.6% (*w*/*w*_0_) ethanol, 2.8 mM NAD^+^, and 25 °C. Data are presented as Mean ± SD (n ≥ 3); * *p* ≤ 0.05, ** *p* ≤ 0.01, and *** *p* ≤ 0.001.

We observed that the *R* values for Reaction 1 were significantly different depending on the YADH and cross-linking agent concentration (Figure 1a). As shown, a 3-fold increase in YADH concentration led to a ~2–4-fold increase in *R* values (Figure 1a; cf. the activity for samples D1–D2 and D3–D4). At the same time, a 10-fold decrease in EDC/S-NHS concentration led to increased *R* values for all enzyme concentrations (Figure 1a; cf. the activity for samples D1 and D2 or samples D3 and D4). It should be pointed out that the nanoparticle concentrations in all cases were 1 × 10^10^ nanoparticles/mL. Thus, the increase in YADH activity with the increase in enzyme concentration (during its conjugation) could indicate that a larger amount of the enzyme could be bound to the MNPs (both covalently and non-covalently). At the same time, we observed a decrease in conjugated YADH activity with an increase in EDC/S-NHS concentration (during enzyme conjugation), indicating the possible modification of active site groups upon binding to MNPs. Thus, changes in YADH activity depended strongly on the amount of bounded enzyme and the possible modification of active site groups. Previously, we showed that the enzyme activity indeed decreased as a result of chymotrypsin binding to GCMNPs (Table S3) [22]. It was, nevertheless, very important for us that both enzymes studied in the work presented were active after all of the synthesis procedures, and we focused on the mechanisms of LFMF action on enzymes in different types of aggregates and did not study the changes in enzyme activity during the formation of aggregates with GCMNPs.

We then studied the effect of LFMF exposure on the activity of YADH in the “dimeric” aggregates with GCMNPs (Figure 1b). It should be noted that there were no effects of LFMF exposure on the catalytic activity of the native (without MNPs) enzyme (Figure S6). We observed YADH down-regulation under exposure to LFMF, and the depths of these changes differed for samples synthesized under various conditions. As shown, exposure to LFMF led to a significant decrease in the enzymatic activity in samples D1 and D2 by 67 ± 9% and 38 ± 2%, respectively. In contrast, the LFMF effect on YADH activity in samples D3 and D4 was insignificant. We posit that the different responses of YADH in the “dimeric” aggregates with GCMNPs upon LFMF exposure were a result of varying the enzyme conjugation conditions (enzyme and cross-linking agent concentrations), which led to different amounts of conjugated enzyme or MNP–enzyme–MNP bonds.

**Figure 2:**
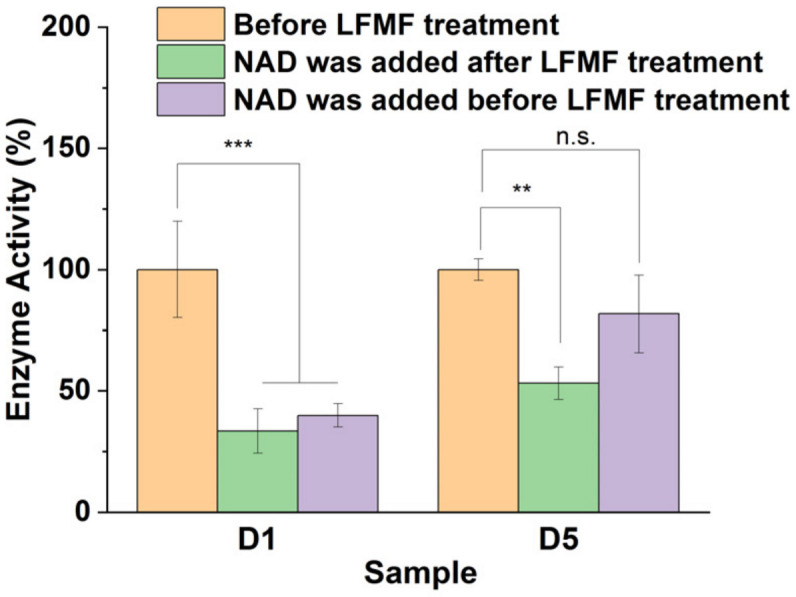
Influence of the presence or absence of a cofactor in the active sites of YADH and FDH in “dimeric” aggregates with GCMNP-LA on the effect of LFMF exposure. NAD^+^ was added before or immediately after LFMF treatment (the same as in Figure 1). Enzymatic activity was measured according to Reactions 1 and 2 for Samples D1 and D5, respectively. The conditions for Reaction 1 are the same as those described in Figure 1. For Reaction 2, 20 mM of Tris-HCl buffer (pH 8.2), 0.3 M of sodium formate, 7.9 mM of NAD^+^, and 25 °C were used. The nanoparticle concentration was 1 × 10^10^ particles/mL. Data are presented as Mean ± SD (n = 3); ** *p* ≤ 0.01 and *** *p* ≤ 0.001. n.s.: not significant.

The effect of LFMF exposure on enzyme activity was also examined for FDH in the “dimeric” aggregates with GCMNP-LA (sample D5) according to Reaction 2. Similarly to YADH in the “dimeric” aggregates, the activity of FDH significantly decreased (by 47 ± 7%) as a result of LFMF treatment (Figure 2). However, this enzyme was not as down-regulated as YADH because the optimal conditions could differ in this case (because we did not vary the enzyme conjugation conditions).

In the case of hydrogenases, the binding of a cofactor in the enzyme’s active site can lead to significant conformational changes in the enzyme’s molecular structure [32,33,34]. Therefore, we studied the impact of cofactor presence or absence in active sites of YADH and FDH in the “dimeric” aggregates with GCMNP-LA on the effect of LFMF treatment (Figure 2, green and purple columns). To measure enzyme activity in this case, we added a cofactor to the enzyme in the “dimeric” aggregates before (green columns) or immediately after (purple columns) LFMF exposure. Surprisingly, YADH and FDH in the “dimeric” aggregates with GCMNP-LA demonstrated different responses to the presence or absence of cofactors. YADH activity decreased after LFMF exposure for both holo- and apo-enzymes (with and without a cofactor, respectively). At the same time, a decrease in FDH activity after LFMF exposure was observed only for the apo-enzyme (without a cofactor). It was previously shown that substrate binding in the active site of dehydrogenases can lead to significant changes in enzyme conformation (switching from an open to a closed form) [32,33,34]. Applying mechanical forces to an enzyme molecule can affect its structure depending on the strength of cofactor-active site interactions [35,36,37], a factor that depends on the binding constant (K_M_). The stronger this interaction, the more difficult it is to affect enzyme structure via mechanical forces. As shown in Figure 2, we observed different responses of YADH and FDH in the “dimeric” aggregates upon LFMF exposure with or without an added cofactor. This finding can be explained by the differences in K_M_ values, which were 170 [38] and 13 µM [39] for YADH and FDH, respectively; therefore, NAD^+^ binding was much stronger in the case of FDH. As a result, we observed a down-regulation for both the apo and holo forms of YADH, and we did not observe a down-regulation for FDH in the holo form.

**Figure 3:**
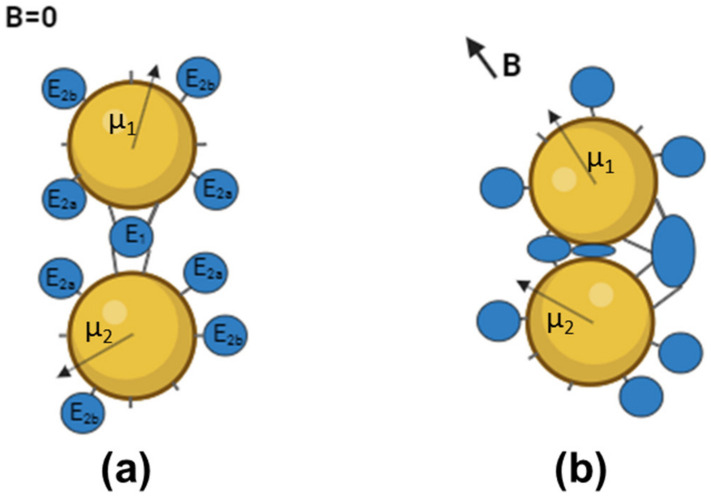
The scheme of the MNM approach for enzyme activity down-regulation. (**a**) The aggregate consists of two MNPs, with magnetic moments µ_1_ and µ_2_, and enzyme molecules bound to the surface of the MNPs. (**b**) The rotational movement of MNPs, tending to orient their magnetic moments along the lines of an external magnetic field *B*, generates four types of deformations, namely, stretching, compression, torsion, and shifting, that affect E_1_ and E_2a_ molecules. E_2b_ molecules are not subjected to mechanical deformation. This figure is a schematic representation and does not reflect MNPs’ or proteins’ relative sizes or MNP heterogeneity. This figure was created on Biorender.com (app.biorender.com).

The observed down-regulation of YADH and FDH activity in the “dimeric” aggregates under exposure to an LFMF was similar to the effect previously observed for ChT in “dimeric” aggregates. These enzymes are from different classes and have very different structures, thus confirming the universality of the MNM approach. We believe that the decrease in YADH and FDH activity in the aggregates resulted from the rotational movement of MNPs under exposure to an LFMF. Figure 3 presents a scheme of such an effect for aggregates consisting of two MNPs and enzyme molecules. There are two enzyme molecule “populations” on the surfaces of MNPs: E_1_, bound to two MNPs simultaneously, and E_2_, bound only to one MNP. During the rotational movement of MNPs in the aggregate under exposure to an LFMF, only E_1_ will express stretching, compression, twisting, and shifting deformations. Because the observed changes in enzyme activity in the “dimeric” aggregates subjected to an LFMF were much greater than 10%, some E_2_ molecules can also be subjected to MNM stimulation.

As shown in Figure 3b, subpopulation E_2a_ (part of E_2_ molecules) could also undergo compression, twisting, and shifting deformations because the two MNPs in an aggregate can vary in size, shape, and magnetic moment [24]. Moreover, the vector of magnetic induction *B* is randomly oriented relative to vector µ. The portion of E_2a_ molecules can reach up to 50% of the total amount of enzyme molecules in an aggregate. Another part of the E_2_ molecules (“subpopulation E_2b_”) cannot be affected by forces generated by MNPs. Note that E_2a_ molecules are close to E_1_ molecules, while E_2b_ molecules are far from E_1_ molecules. Thus, the maximal amount of enzyme molecules affected by mechanical forces produced by MNPs cannot exceed ~50–60%. This value is in good agreement with the maximal decrease in enzyme activity in the dimeric aggregates under exposure to LFMF observed in this and previous work. Notably, the presence of non-covalently-bound enzyme molecules should increase the E_2b_ portion, thereby decreasing the efficiency of the LFMF effect on enzyme activity. As shown in Figure 1b, the down-regulation of YADH in samples D1 and D2 was much greater than that in samples D3 and D4, while the YADH concentrations were greater in samples D3 and D4 (mainly due to non-covalently-bound enzyme molecules). We posit that in the case of samples D3 and D4, greater enzyme concentrations led to an increase in the E_2b_ portion, and, as a result, the observed effect of LFMF on enzyme activity was negligible.

Thus, the observed effects of an LFMF on the catalytic activity of enzymes in “dimeric” aggregates with MNPs significantly depend on the aggregate structure and the presence of non-covalently bound enzymes. Even though all of the produced aggregates with YADH were of the “dimeric” type, only the samples with low enzyme concentrations responded to LFMF treatment. Consequently, we believe that the selection of enzyme conjugation conditions should be based on the impact of an LFMF on enzyme kinetics in aggregates and not only on observing hydrodynamic parameters. Thus, based on the experimental data, we can define major patterns for choosing the conditions of an enzyme binding to MNPs’ surface, allowing for an increase in the portion of “dimeric” aggregates and a reduction in the portion of “monomeric” aggregates, thereby increasing the effect of an LFMF:

- The optimization of the enzyme concentration during binding to the MNPs’ surface (at a constant MNPs concentration). An increase in enzyme concentration naturally leads to the enzyme’s increased activity in an aggregate. At the same time, an increase in enzyme concentration boosts non-covalent enzyme binding, which leads to a decrease in LFMF effectiveness.
- The optimization of a cross-linking agent’s concentration for the binding of an enzyme to MNPs’ surface (at a constant enzyme concentration). An increase in the EDC/S-NHS concentration naturally suppresses the enzyme activity in aggregates due to the possible modification of active site groups. At the same time, an increase in the EDC/S-NHS concentration can enhance LFMF effectiveness by promoting the formation of “dimeric” aggregates.

### 3.4. Up-Regulation of FDH in “Monomeric” Aggregates

To study the influence of hydrodynamic forces, we evaluated the effect of an LFMF on the enzyme (FDH) in “monomeric” aggregates with GCMNP-PEG. Although such forces are much weaker than “contact” forces produced by “dimeric” aggregates subjected to an LFMF, the latter can affect weak interactions in enzyme molecules [40]. Previously, we showed [23] that in the case of “dimeric” aggregates, the down-regulation of enzyme activity under LFMF treatment was much more pronounced with short low-molecular-weight ligands as a result of a more effective transfer of contact forces from MNPs to the enzyme. In the case of “monomeric” aggregates, the hydrodynamic force *F_HD_* applied to enzyme molecules on the surfaces of GCMNPs can be determined using the Stokes equation:

$$F_{HD}=6\pi \eta R_{E}R_{HD}\dot{\varphi },$$

where *η* denotes the viscosity of the media, *R_E_* denotes the radius of the enzyme molecule, *R_HD_* denotes the hydrodynamic radius of the aggregate, and *φ* denotes the angle between the MNP magnetic moment vector and the direction of the LFMF. The hydrodynamic diameter of GCMNP-PEG is higher than that of GCMNP-LA (Table 1); therefore, one can expect a higher value of *F_HD_* applied to enzyme molecules under LFMF treatment in the case of GCMNP-PEG than that for GCMNP-LA.

**Figure 4:**
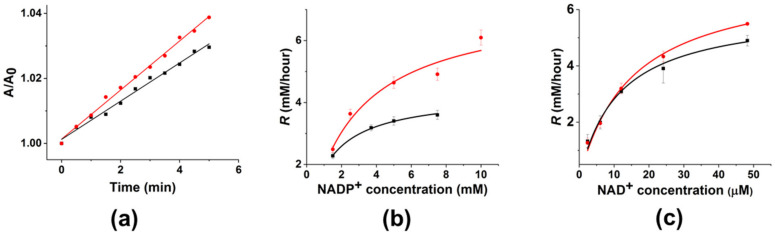
The effect of three cycles of a “pulsed” LFMF (*f* = 50 Hz, *B* = 140 mT, Pulse/Pause = 1 min/30 s) on the FDH kinetics (Reaction 2) in “monomeric” aggregates with GCMNP-PEG. (**a**) Kinetic curve using NADP^+^ (7.5 mM) as a cofactor. (**b**,**c**) The dependence of initial reaction rates (*R*) on (**b**) NADP^+^ and (**c**) NAD^+^ concentration. Data on the periods before and after LFMF treatment are presented in black and red, respectively. A 20 mM Tris-HCl buffer (pH 8.2), 0.3 M of sodium formate, and 25 °C were the conditions used. The nanoparticle concentration was 3.8 × 10^10^ particles/mL. Data are presented as Mean ± SD (n = 3).

The soybean FDH used in this work is capable of catalysis with two cofactors, NAD^+^ and NADP^+^, because their structures are similar, except for the phosphate group in NADP^+^; therefore, we studied the effect of an LFMF on enzyme activity for both of these cofactors. In contrast to FDH down-regulation in the “dimeric” aggregates, we observed enzyme up-regulation under LFMF treatment in these experiments; however, the magnitude of this effect varied for different cofactors. As shown in Figure 4a, the kinetic curve rises faster (up to 40%) under an LFMF than in the absence of an LFMF. Figure 4b,c depict the dependence of initial rates according to Reaction 2 on the concentrations of NADP^+^ and NAD^+^, respectively. As shown, all of the curves are in good agreement with typical Michaelis–Menten dependence. However, the effect of the LFMF was much higher for NADP^+^ than for NAD^+^. Note that the maximal up-regulation of FDH in the “monomeric” aggregates was 40 ± 10% using NADP^+^ (7.5 mM).

**Figure 5:**
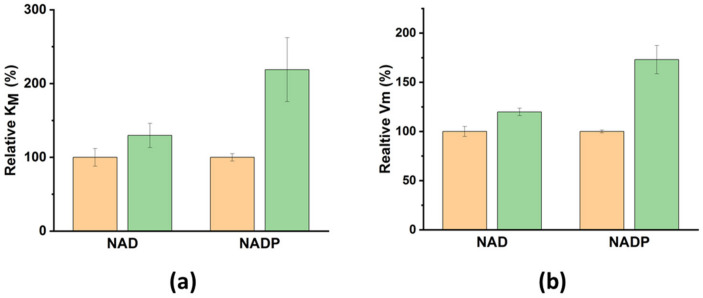
The effect of LFMF treatment on (**a**) relative K_M_ and (**b**) relative V_m_ for FDH in “monomeric” aggregates. Data for the periods before and after LFMF treatment are presented in orange and green, respectively. All other conditions are the same as those in Figure 4.

For a more detailed analysis of FDH up-regulation in the “monomeric” aggregates, we calculated the relative values of V_m_ and K_M_ for NAD^+^ and NADP^+^ with (orange columns in Figure 5) and without (green columns in Figure 5) LFMF. As shown, there are almost no changes in K_M_ and V_m_ for NAD^+^ used as a cofactor. At the same time, the LFMF treatment led to a significant increase in K_M_ and V_m_ for NADP^+^ by 120 ± 40% and 70 ± 10%, respectively. Thus, the effect of the LFMF on FDH in the “monomeric” aggregates led to significant enzyme up-regulation using only NADP^+^, potentially indicating possible changes in enzyme selectivity toward this cofactor. We have previously shown that the rotational movement of “monomeric” aggregates under the action of an LFMF generates hydrodynamic forces, which could not significantly affect an enzyme’s secondary structure. However, such hydrodynamic forces can change the electrostatic interactions in polymers on the surface of rotating MNPs [40]. In our case, we posit that hydrodynamic forces can alter the hydrogen bond network and disrupt weak Van der Waals forces and electrostatic interactions in enzyme molecules. This effect can be sufficient for fine-tuning the active site of FDH in “monomeric” aggregates to achieve more efficient catalysis toward one of the cofactors due to the binding constant of an enzyme.

Previously, we showed that an LFMF had no effect on the activity of the hydrolytic enzyme ChT in “monomeric” aggregates with GCMNPs [21]. In this study, unexpectedly, in the case of FDH, we found a strong difference between the effects of an LFMF on the enzyme in “dimeric” aggregates with or without an added cofactor (Figure 2). No such difference was found in the case of YADH. Because hydrodynamic forces are much weaker, we neither expected nor saw any effects on YADH in the “monomeric” aggregates under LFMF action. However, for *Glycine max* FDH, which has both NAD^+^ and NADP^+^ activity, of which the latter is orders of magnitude lower, we were expecting sensitivity even to a weak effect on enzyme structure and a possible difference in cofactor selectivity. Indeed, we revealed strongly different responses in enzyme activity after the application of an LFMF to FDH in the “monomeric” aggregates toward NAD^+^ and NADP^+^.

## 4. Conclusions

Herein, the up- and down-regulation of YADH and FDH were studied. Under exposure to an LFMF, MNPs can inflict mechanical forces and deformations on enzyme molecules that are attached to MNPs’ surface. The nature and magnitude of such forces vary depending on the MNP-enzyme aggregate structure. We synthesized GCMNPs capable of rotational movement under LFMF treatment and used them to produce distinct types of GCMNP–enzyme aggregates. We have shown that the activity of YADH and FDH in “dimeric” aggregates decreases under exposure to an LFMF (enzyme down-regulation), while the activity of FDH in “monomeric” aggregates increases (enzyme up-regulation).

We observed various YADH activities and magnitudes of the LFMF effect depending on the enzyme and cross-linking agent concentration used for “dimeric” aggregate synthesis. Based on our experimental data, we gave recommendations for enzyme binding to MNPs that increase the LFMF effect. Moreover, we observed that the down-regulation of YADH and FDH in “dimeric” aggregates varied depending on the presence or absence of a cofactor in the enzyme active site; such an effect resulted from the difference in cofactor binding efficiency for the two enzymes. We observed enzyme up-regulation for “monomeric” aggregates with FDH, and the magnitude of this effect was much higher when using NADP^+^ as a cofactor compared to when NAD^+^ was used. The kinetic parameters (K_M_ and V_m_) for FDH were determined. We found a substantial increase in both K_M_ and V_m_ only in the case of NADP^+^. We posited that hydrodynamic forces generated by the rotational movement of MNPs in LFMF fine-tune the enzyme active site for catalysis toward one of the cofactors. We believe that such up- and down-regulation of enzymes can find applications in industrial processes based on enzymatic reactions.

## Data Availability

The original contributions presented in this study are included in the article and Supplementary Materials; further inquiries can be directed to the corresponding author.

## Supplementary Materials

The following supporting information can be downloaded at https://www.mdpi.com/article/10.3390/nano14050411/s1, Table S1. The YADH:GCMNPs and EDC/S-NHS:YADH ratios used for enzyme conjugation; Figure S1: TEM micrographs of (a) synthesized iron oxide magnetic cores and (b) GCMNPs; Table S2. Mössbauer parameters obtained from fitting the experimental spectra; Figure S2. Mössbauer spectrum of iron oxide MNPs at 300 K; Figure S3. Characterization of GCMNPs modified with lipoic acid (LA) and SH-PEG_5000_-COOH; Figure S4. Distribution of hydrodynamic sizes for samples D1–D5 obtained via NTA; Table S3. Changes in ChT activity as a result of binding to GCMNPs; Figure S5. Characterization of GCMNP-PEG_5000_-COOH and GCMNP-PEG-FDH via (a) NTA and (b) TEM; Figure S6. Impact of LFMF on the enzymatic activity of native (unbound to GCMNPs) YADH and FDH.
