# Supplementary material for: Up- and Down-Regulation of Enzyme Activity in Aggregates with Gold-Covered Magnetic Nanoparticles Triggered by Low-Frequency Magnetic Field
Source: Nanomaterials (Basel). 2024 Feb 23;14(5):411. doi: 10.3390/nano14050411 (PMC10935337; doi:10.3390/nano14050411)
Supplement: Supplementary file 1 [file nanomaterials-14-00411-s001.zip › nanomaterials-2852425-supplementary.pdf]

Supplementary Materials

Up- and Down-Regulation of Enzyme Activity in Aggregates with Gold-Covered Magnetic Nanoparticles Triggered by Low-Frequency Magnetic Field

Maxim M. Veselov [1], Maria V. Efremova [1,2], Andrey N. Prusov [3] and Natalia L. Klyachko [1,*]

[1] School of Chemistry, Lomonosov Moscow State University, Moscow 119991, Russia; veselov.mac@gmail.com (M.M.V.); m.efremova@tue.nl (M.V.E.)
[2] Department of Applied Physics and Science Education, Eindhoven University of Technology, 5600 MB Eindhoven, The Netherlands
[3] A.N. Belozersky Institute of Physico-Chemical Biology, Lomonosov Moscow State University, Moscow 119992, Russia; prusov@genebee.msu.su
* Correspondence: klyachko@enzyme.chem.msu.ru

Enzymes Conjugation

To synthesize "dimeric" aggregates with YADH, 0.5 ml of GCMNPs-LA was typically used. The suspension of GCMNPs-LA (24.3×10^{10} particles/ml) containing EDC and S-NHS ($0.36\text{-}7.8 \times 10^{-4}$ M) was stirred for 15 minutes at 4°C, followed by centrifugation at 6000xg for 10 minutes at 4°C. The sediment was resuspended in 0.5 ml of 5 mM phosphate buffer (pH 6.0) contained YADH ($0.4\text{-}1.2 \times 10^{-5}$ M), the reaction mixture was stirred for 1.5 hours at 4°C and then GCMNPs-YADH conjugates were separated by centrifugation (3 times, 6000xg, 4°C) and resuspended in 0.5 ml of 20 mM Tris-HCl buffer (pH 8.2). YADH: GCMNPs and EDC: YADH ratios are presented in Table S1. The EDC: S-NHS Ratio was 1:1 for all samples.

Table S1. The YADH: GCMNPs and EDC/S-NHS: YADH ratios used for enzyme conjugation. The EDC:S-NHS ratio was 1:1 in all cases. GCMNP-LA concentration 24.3×10^{10} particles/ml determined by NTA. A molecular weight equal to 150000 g/mol was used to determine the amount of YADH molecules (Protein Data Bank Code 4W6Z).

Sample	YADH: GCMPNs	EDC/S-NHS: YADH
D1	10000:1	200:1
D2	10000:1	20:1
D3	30000:1	65:1
D4	30000:1	6.5:1

To synthesize "dimeric" aggregates with FDH, 3 ml of GCMNPs-LA was mixed with 150 µg EDC and 180 µg S-NHS, stirred for 15 min at 4°C followed by centrifugation at 6000xg for 10 minutes at 4°C. The sediment was resuspended in 2.8 ml of 20 mM Tris-HCl buffer (pH 8.2), after which 0.2 ml of FDH suspension was added, and the reaction mixture was stirred for 1.5 hours at 4°C. The purification step was similar to YADH conjugation.

To synthesize “monomeric” aggregates with FDH, 1 ml of GCMNP-PEG$_{5000}$-COOH was mixed with 2 mg EDC and 2.2 mg S-NHS and stirred for 15 min at 4°C followed by centrifugation at 6000xg for 10 minutes at 4°C. The sediment was resuspended in 1 ml of 20 mM Tris-HCl buffer (pH 8.2), after which 0.25 ml of FDH suspension was added, and the reaction mixture was stirred for 1 hour at 4°C. The purification step was similar to the one in the protocol for “dimeric” aggregates synthesis.

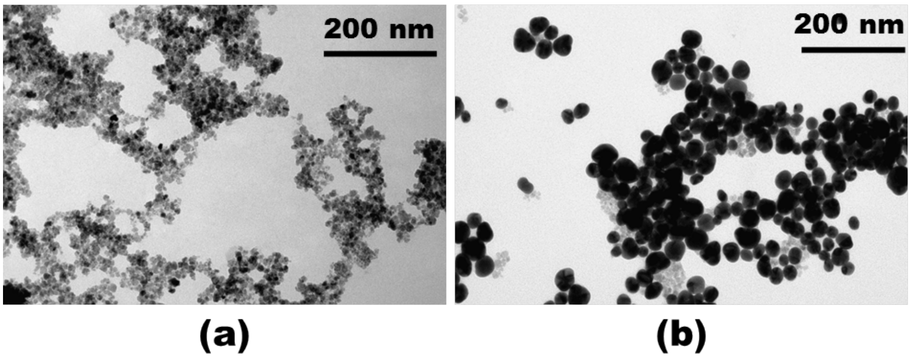

Figure S1. TEM micrographs of (**a**) synthesized magnetic cores of iron oxide and (**b**) GCMNPs. Our group has previously published these images in [1].

Mössbauer Spectroscopy

(We have previously published this data, cf. ref. [1]) Table S2 summarizes the parameters extracted from fitting the Mössbauer spectra of iron oxide nanoparticles (Figure S2). d_s – isomeric shift, D - quadrupole splitting, H_{eff} – effective magnetic field. The corresponding errors are 5 kOe for H_{eff}, 0.02 mm/s for d_s, 0.01 mm/s for D, and 0.6% for determining component area S.

A feature of the Mössbauer spectra of nonstoichiometric magnetite is the distribution of the electron exchange between Fe^{3+} and Fe^{2+} ions in the octahedral positions in the presence of vacancies. At the same time, some of the Fe^{3+} ions that are not involved in such exchanges because of the Fe^{2+} ions deficit form a spectrum that is not distinguishable from a Mössbauer spectrum of Fe^{3+} ions in the tetrahedral positions. The accuracy of Mössbauer spectroscopy for such fitting is about 3%. The parameter of vacancies x can be calculated as [36,37] :

$$x = \frac{1{,}88\frac{S_A}{S_B} - 1}{4{,}7 + 5{,}64\frac{S_A}{S_B}}$$

where S_A and S_B are the relative Mössbauer spectral weights of Fe^{3+} ions in tetrahedral and octahedral sites, respectively. The fitting delivers $\frac{S_A}{S_B}$ = 1.39 resulting in x = 0.129. Thus, the $Fe_{3-x}O_4$ stoichiometry can be written as $Fe^{2+}_{0,61}Fe^{3+}_{2,26}O_4$ with a corresponding ratio of Fe^{2+}/Fe_{total} = 0.21.

Table S2. Mössbauer parameters obtained from fitting the experimental spectra. We have previously published this data in [1].

Name	d_s, mm/s	D, mm/s	H_{eff}, kOe	Area S, %	Component	Total area of Fe^{3+} ions in tetrahedral site S_A, %	Total area of Fe^{3+} ions in octahedral sites S_B, %
Sextet 1	0.2933	0.2960	447.49	11.23	Fe^{3+} (A)	50.88	36.50

Sextet 2	0.4163	0.3192	446.41	9.79	Fe^{3+} (B)
Sextet 3	0.3193	0.0990	404.78	10.68	Fe^{3+} (A)
Sextet 4	0.2989	0.1502	373.31	9.41	Fe^{3+} (A)
Sextet 5	0.7604	0.7532	333.60	9.24	Fe^{3+} + Fe^{2+} (B)
Sextet 6	0.2694	0.2142	283.44	7.84	Fe^{3+} (A)
Sextet 7	0.3889	0.2778	191.79	11.53	Fe^{3+} (B)
Doublet 1	0.2314	0.9309		11.72	Fe^{3+} (A)
Doublet 2	0.8030	1.3255		5.94	Fe^{3+} + Fe^{2+} (B)

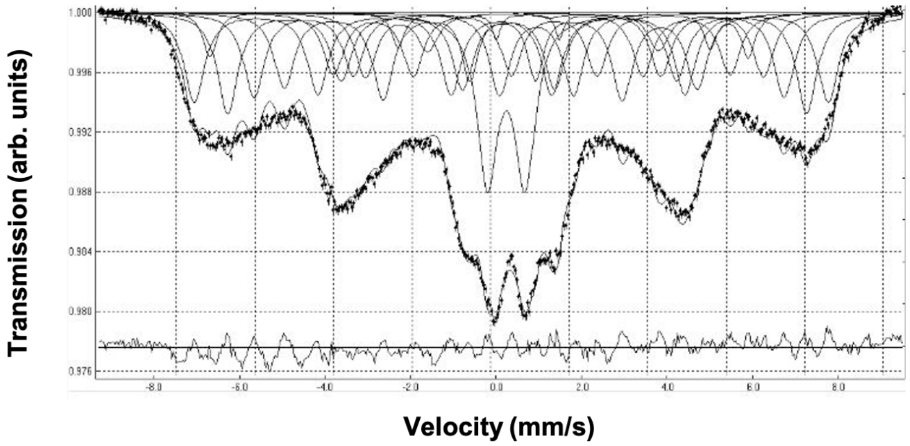

Figure S2. Mössbauer spectrum of iron oxide MNP at 300 K. A bold line represents the experimental spectrum simulated with 7 sextets and 2 doublets (thin lines) corresponding to various combinations of Fe^{2+} and Fe^{3+} signals in tetrahedral and octahedral positions. The spectrum at the bottom corresponds to the difference between the experiment and the simulation. We have previously published this data in [1].

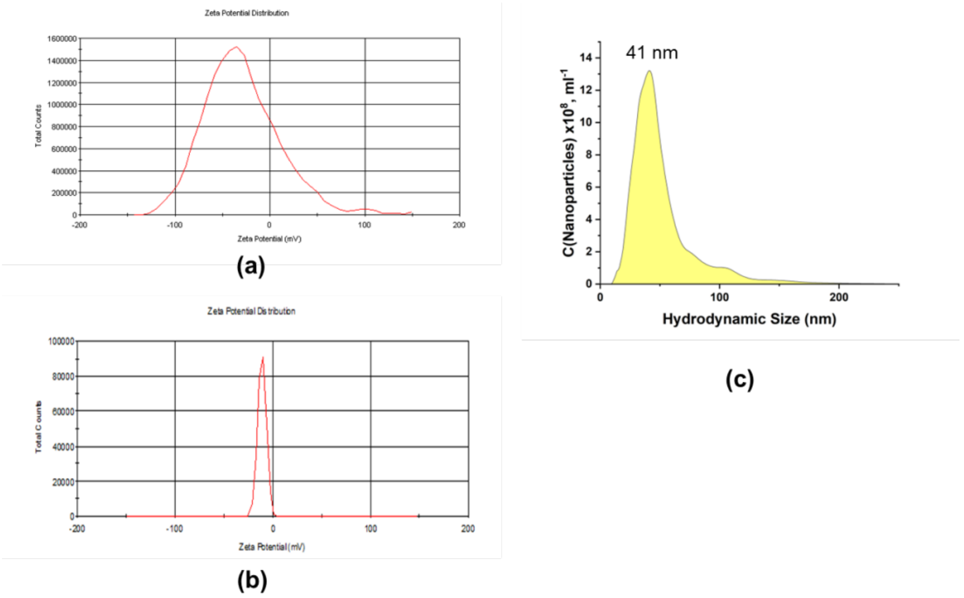

Figure S3. Characterization of GCMNPs modified with lipoic acid (LA) and SH-PEG_{5000}-COOH. (**a, b**) Zeta potential of (**a**) GCMNP-LA and (**b**) GCMNP-PEG obtained by DLS. (**c**) distribution of hydrodynamic sizes of GCMNP-LA obtained by NTA.

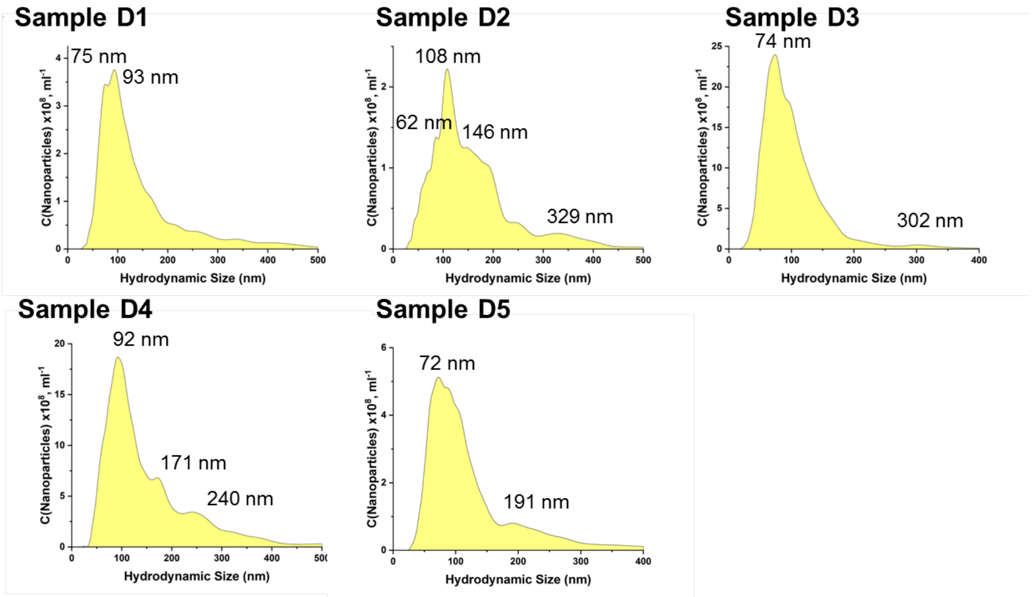

Figure S4. Distribution of hydrodynamic sizes for Samples D1-D5 obtained via NTA.

Table S3. Changes of ChT activity as a result of binding to GCMNPs. We have previously published this data in [1].

Sample	k_{cat}/K_M, 1/(μM*min)
ChT	74
GCMNP-ChT	38

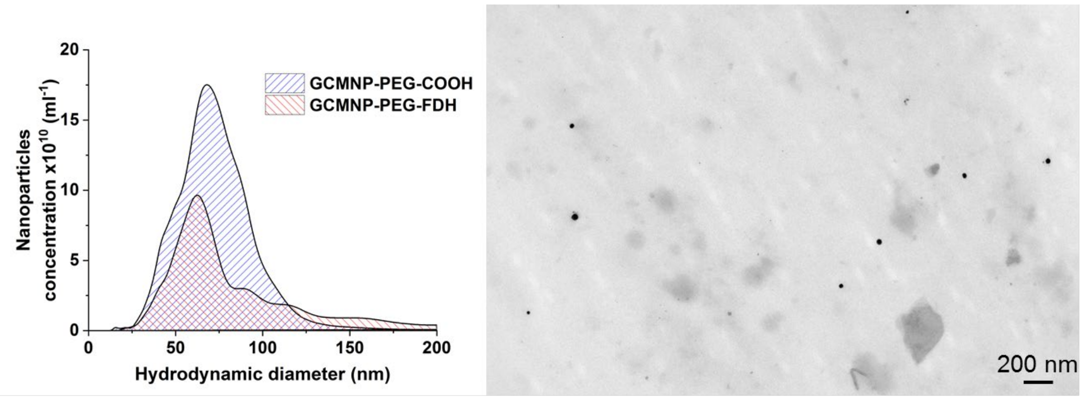

(a) **(b)**

Figure S5. Characterization of GCMNP-PEG$_{5000}$-COOH and GCMNP-PEG-FDH via (**a**) NTA and (**b**) TEM.

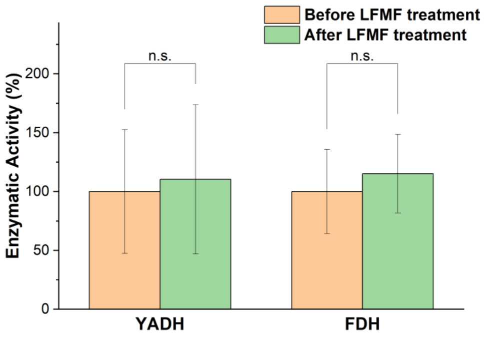

Figure S6. Impact of LFMF on the enzymatic activity of native (unbound to GCMNPs) YADH and FDH. The conditions of the enzyme activity study and LFMF exposure are the same as in Figures 1 and 2.

References

1. Veselov, M.M.; Uporov, I.V.; Efremova, M.V.; Le-Deygen, I.M.; Prusov, A.N.; Shchetinin, I.V.; Savchenko, A.G.; Golovin, Y.I.; Kabanov, A.V.; Klyachko, N.L. Modulation of α-Chymotrypsin Conjugated to Magnetic Nanoparticles by the Non-Heating Low-Frequency Magnetic Field: Molecular Dynamics, Reaction Kinetics, and Spectroscopy Analysis. *ACS Omega* **2022**, *7*, 20644-20655, doi:10.1021/acsomega.2c00704.
